# *“The sun keeps rising but darkness surrounds us”:* a qualitative exploration of the lived experiences of women with obstetric fistula in Ethiopia

**DOI:** 10.1186/s12905-019-0732-3

**Published:** 2019-02-26

**Authors:** Misganaw Animut, Abebe Mamo, Lakew Abebe, Million Abera Berhe, Shifera Asfaw, Zewdie Birhanu

**Affiliations:** 1 Onchocerchiasis and Lymphatic Filariasis Elimination Project in Awi and Metekel Districts, Carter Center Ethiopia, Bahir Dar, Amhara Regional State Ethiopia; 2 Department of Health, Behavior and Society, Institute of Health, Jimma University, PO.Box: 378, Jimma, Ethiopia; 3 School of Nursing and Midwifery, Jimma University, Jimma, Ethiopia

**Keywords:** Lived experience, Obstetric fistula, Ethiopia

## Abstract

**Background:**

Obstetric fistula is a hole between the vagina and bladder, and/or between the vagina and rectum, triggered by prolonged obstructed labor. The World Health Organization has estimated that at least 50,000 to 100,000 cases of obstetric fistula occur every year, and that over two million women with obstetric fistula in developing countries remain untreated. Research on women’s lived experiences of obstetric fistula is limited. This study aimed to explore the lived experience of women with obstetric fistula at Bahir Dar Hamlin Fistula Center, Amhara Regional State, Ethiopia.

**Methods:**

A qualitative study design, drawing from a phenomenological approach, was employed to explore the lived experience of purposively-selected sample of ten women with obstetric fistula. In-depth interviews were conducted in the local language (Amharic) using an interview guide. Interviews were transcribed and translated into English, and transcripts were entered as primary documents into Atlas.ti 7 software. Thematic categories were identified, and transcripts were coded accordingly.

**Results:**

Participants perceived that the contributing factors to obstetric fistula were: instrument-assisted delivery; inappropriate physical examination and care; early marriage; and long duration of labour. As a result of obstetric fistula, the patients suffered from uncontrolled dripping of urine and/or faeces (and associated offensive odours), ostracization by their family and community members, and feeling hopeless and isolation from the community. Patients used different coping mechanisms, including frequent washing of clothes and changing of underwear; they also expressed that they preferred to be alone.

**Conclusion:**

Women with obstetric fistula experienced urine incontinence and associated bad odour; social and psychological problems like isolation, divorce and fears were commonly reported. Our findings from perspectives of Ethiopian setting suggest that integrated services for women with obstetric fistula are warranted, including physical therapy, psychological support and social reintegration.

**Electronic supplementary material:**

The online version of this article (10.1186/s12905-019-0732-3) contains supplementary material, which is available to authorized users.

## Background

Obstetric fistula is a condition characterized by a hole in the bladder and/or rectum that opens into the vagina, through which urine or faeces leak uncontrollably. It is typically acquired when there is a delay to intervene in a prolonged and obstructed labor [1]. Fistula tends to occur more often in places where access to and use of obstetric care is limited. It affects remote regions where there is strong stigmatization of the condition and its complications [2].

Obstetric fistula affects about two million women in developing countries, and an estimated 50,000 to 100,000 new cases occur each year [3]. The condition is prevalent in sub-Saharan Africa and Asia. In Sub-Saharan Africa alone, an estimated 30,000 to 130,000 women giving birth develop obstetric fistula each year [4]. In Ethiopia, an estimated 9000 women develop obstetric fistula each year, and 1200 of these women are treated by Hamlin Fistula Hospital. Almost all cases of fistula seen at Hamlin Fistula Hospital were caused by obstetric complications related to prolonged and obstructed labor [5–7].

Obstetric fistula has many consequences: about 93% of women with obstetric fistulas had a stillbirth and 97% develop mental health problems such as depression. Among those who experienced depression, 54% reported suicidal ideation. Further, 68% had no living children, nearly 54% were divorced, 13% were not allowed to eat with family members and 41% did not belong to any community association [7]. Obstetric fistula constitutes a severe threat to the health of adolescent women in particular [5].

While the area of maternal health has improved in Ethiopia in recent years, women still suffer from maternal health problems, with a maternal mortality ratio of 412 deaths per 100,000 live births. Further, utilization of maternal health services is low, with only a third of pregnant women attending four antenatal care visits, and only a quarter of women giving birth at health facilities [8]. This implies that nearly three quarters of births occur in the home without a skilled delivery assistant; women delivering at home may be at increased risk for prolonged and obstructed labor, as well as resulting complications, including obstetric fistula [1, 8].

Obstetric fistula has serious social and physical health implications for women in Ethiopia. To date, researchers have mainly focused on assessing awareness and quantifying the magnitude of the problem. The recent Ethiopian Demographic and Health Survey in 2016, for instance, reported on awareness and prevalence of obstetric fistula; however, it did not collect data about the perceived causes of obstetric fistula, nor its social, physiological and physical consequences. There is a lack of research about how women experience obstetric fistula and its implications on their daily lives. Therefore, the current study addressed those issues by exploring the lived experiences of women with obstetric fistula in Amhara Regional State of Ethiopia, where home delivery and early marriage are common.

## Methods

Qualitative methodology, increasingly used in the field of public health, was chosen for this study due to its appropriateness in answering the research questions [9]. This research drew from a descriptive phenomenological qualitative study design; to explore the lived experience of women with obstetric fistula at Bahir Dar Hamlin Fistula Centre in Bahir Dar, Amhara Regional State. A descriptive phenomenological qualitative design, developed by Edmund Husserl, focuses on the experience – the *what* and the *how* of the phenomena – and calls upon the researchers to bracket prior opinions and biases. Later, Martin Heidegger developed interpretive phenomenological approach. He recommended that a researcher cannot investigate ‘things in their appearing’ to identify their essences while remaining objective and unbiased. However, most researchers prefer the classic approach for phenomenology [10]. They believed that it is possible to bracket out one self from the phenomena. The current research used a descriptive phenomenological approach whereby researchers employed bracketing to gain perspective on the phenomena. This research reported the experience of living with obstetric fistula (i.e. the “what”) and the experience of becoming an obstetric fistula patient (i.e. the “how” it is experienced). The use of in-depth interviews with experienced data collectors permitted the exploration of aspects of individual lived experience that may not be openly discussed in a normal circumstance due to the culturally-sensitive nature of the subject [11, 12].

### Study setting and period

Data were collected in November and December 2016 at the Bahir Dar Hamlin Fistula Centre. The Centre, established in 2005, is a branch of the Addis Ababa Hamlin Fistula Hospital. It is located 340 miles north-west of Addis Ababa, near the Felegehiot Hospital (a large public hospital serving the Amhara Region). It serves the northwest part of the Amhara Region, and the entire Benshangul-gumz Region. The Bahir Dar Hamlin Fistula Centre provides surgical repair and psychological rehabilitation services for women admitted with obstetric fistula. The facility has 44 beds. The Center works with community health agents and health care providers from different districts (woredas) in the catchment area and supports community outreach to raise awareness about obstetric fistula prevention and strengthen referral linkages from community health facilities.

#### Study population

Participants were purposively selected from women admitted with obstetric fistula to Bahir Dar Hamlin Fistula Centre. We included women who came to the Centre for the purpose of recurrence of the problem after one or more surgical treatments; we did not include women who were not admitted or who were unable to participate in an in-depth interview due to serious illness or due to the pain after surgery. New participants were approached until data saturation had been reached (until no new ideas were emerging).

### Sampling technique

Thirty-six women attended the Centre during the study period. The researchers conducted an initial rapid assessment with each woman to learn their diagnosis of obstetric fistula and their frequency of visiting the Centre for surgery – information that was obtained from the information on their health card. Ten women with obstetric fistula and treatment failures were selected for the current study.

### Data collection technique and analysis

Data were collected through in-depth interviews, using open-ended questions. The participants’ emotions and other nonverbal communication were recorded as field notes. In depth interviews were conducted one-to-one in a quiet and private place to respect patient confidentiality. The time of interview was scheduled based on mutual agreement with the participants.

The in-depth interview guide was developed by the principal investigator, Misganaw Animut (MA), after reviewing relevant literature and consulting similar guidelines (Additional file 1). The interview guide covered socio-demographic variables, reproductive history, physical health, and psychosocial lived experiences of women with obstetric fistula. Participants were asked about: their general health condition before having obstetric fistula; their perceptions about why they experienced obstetric fistula; their pregnancy and marital history; their labor and delivery history; their physical health condition following obstetric fistula; and their experiences related to family, husband, relatives and other social groups. Participant psychological feelings and coping strategies were also explored, and participants were probed for additional contextual information about their experiences. All participants agreed to be audio-taped and interviews lasted between 60 and 78 min.

The in-depth interview guide was prepared first in English and then translated into Amharic and retranslated back to English to check for consistency. The in-depth interview guide was pre-tested among obstetric fistula patients following treatment in the Gynecology and Obstetrics ward at Jimma University Specialized Hospital to assess the clarity of the questions and to measure the length of time for the full interview. Data were obtained in the form of digital audio records, and field notes, including non-verbal expressions. During the interview, the researcher asked questions, took notes, and recorded participants’ non-verbal expressions. Data collection and analysis occurred simultaneously. Audio recordings of the interview and the field notes were transcribed. The researchers translated all Amharic version transcripts into English prior to analysis.

All interview transcripts were read multiple times by Misganaw Animut (MA), Abebe Mamo (AM), and Shifera Asfaw (SA), and the preliminary code guide was applied to all transcripts independently. Discrepancies in the application of the code guide were reviewed and resolved. Following these processes, a final version of the code guide was developed through consensus by MA, AM and SA. This exercise ensured the coders had a common understanding of the code guide and its application. The resulting code guide allowed us to capture all major ideas raised by the participants. Coding was done by Atlas.ti version 7 software. Quotations for selected codes were generated using the Atlas.ti Code Manager/Output feature. Finally, the codes were sorted into relevant categories, and the main themes and categories were identified (Additional file 2). Significant quotations were clustered into themes, and then used to explain participant experiences and the context that influenced how the participants experienced the phenomenon.

#### Trustworthiness

To promote credibility, the interviews were open ended and respondents were encouraged to answer the questions in an uninhibited manner, while being guided to remain focused on the topic of interest. Before transition to the next statement, the interviewer read back the summary of the questions and subsequent probes were emerged in the preceding section of the in-depth interview guide.

Detailed field notes were kept throughout the research process, providing thick description of the subject matter. Researchers employed bracketing methods, self-monitoring to achieve objectivity to minimize their bias and the risk of reactivity. “Bracketing out” techniques were used by researchers to limit the influence of their preconceived ideas about the study; this was done by adopting an open-ended interview design that followed the cues from participants. An independent researcher who was not part of the study and who had a general understanding of the nature of the study reviewed the interview responses, insights and analysis. This process helped us to get impartial feedback on the research process.

## Results

### Socio-demographic characteristics and reproductive history

Ten participants who were admitted to Hamlin Fistula Centre, Bahir Dar were interviewed (Table 1). The mean age of the participants was 33.8 years. The mean ages of participants at first time of sexual intercourse and first pregnancy were 14.3 years and 17.2 years, respectively. All participants reported that they did not know their husband before marriage and that they had experienced genital cutting during childhood. Four of the participants could read and write while six had no formal education. Five of the study participants were divorced after the onset of obstetric fistula. All participants were Amhara by ethnicity, Ethiopian Orthodox by religion, and farmer by occupation. All resided in rural areas of Amhara Region.

**Table 1:** Background characteristics and reproductive history of participants, Hamlin Fistula Centre Bahir Dar, Ethiopia, December 2016

Characteristic		Value
Mean age		33.8 years
Mean age of first intercourse		14.3 years
Mean age of first pregnancy		17.2 years
Education	No education	6 participants
	Can read and write	4 participants
Occupation		All were farmers
Marital status	Married	5 participants
	Divorced	5 participants
Pregnancy outcome during onset of obstetric fistula	Live birth	2 participants
	Still birth	8 participants
Mean parity		2.6 children
Mean delay for surgery		12.6 years
Number of previous operations	None	3 participants
	One	7 participants
	More than one	0 participants

Study participants had lived with obstetric fistula for an average of 12.6 years before seeking medical treatment. Seven of the participants had undergone surgical treatment before the current visit and failed to be cured.

### Theme 1: Perceived causes of obstetric fistula

Women with obstetric fistula were interviewed about how they came to seek care, and their perceptions about the causes for the onset of the problem. Instrument-assisted deliveries, inappropriate assistance of health workers during delivery, early marriage, prolonged labor, were reported as causes of or contributing factors to obstetric fistula.

### Instrument-assisted delivery

Instrument-assisted delivery, involving the use of forceps and vacuum, are important intervention used by health workers to prevent maternal mortality related to prolonged labor. Some of the participants perceived that instrument-assisted delivery could results in the onset of obstetric fistula. One reason why study participant held this belief might be that health care workers did not adequately explain to pregnant women about the importance of these instruments for assisting with delivery. Health workers have an opportunity to explain the danger signs during pregnancy and delivery while women attend antenatal care visits, but often did not take the time to do so. For example, one of the study participants, a 30-year-old woman who perceived that instrument-assisted delivery caused obstetric fistula explained:

*“I had a healthy pregnancy and follow antenatal care at least for three times. My labor started at midnight and went to the health center in the morning. I didn’t face much problem during labor. Nonetheless, the doctor inserted material inside of my vagina. Then, I became distended. I lost my baby and become unable to control my urine.”*

#### Inappropriate physical examination and cares

Other participants perceived the onset of obstetric fistula to stem from health workers performing a physical examination and inappropriate assistance. They felt that women who come from rural area and who had poor personal hygiene and low educational status did not get appropriate treatment from health workers. They explain that, as a result, patients may develop negative attitudes towards health workers which may result in underutilization of health services. The current study participants perceived that health workers are a source of the problem rather than a solution for their sickness. One participant, a 35-year-old woman, reported having had prolonged labor pain at home for four days. She had an abnormal foot presentation of the fetus, and a craniotomy delivery. She perceived the cause of obstetric fistula to be linked to the hands of health workers, which she believed caused a tearing of her uterus:

*“I had a healthy pregnancy, but during laboring, the baby first presented with legs. I pushed the baby for three days at home but failed to give birth. Then, I was heading to health facility. The doctor inserted her hand to my vagina and pull out the baby. The baby found dead inside of my womb. Finally, they distract the head of the baby and pulled out the ruminant body. The doctor’s hand caused tearing of my urine tube and I became an obstetric fistula patient.”*

Another participant, a 48-year-old woman, also perceived that health workers provided inappropriate care at a health facility which resulted in obstetric fistula:

*“My labor started at home and stayed for two days. The labor pain becomes serious and the baby could not come out, finally my neighbors took me to hospital. At the hospital the female doctor inserted her hand into my womb and pull out the baby. The baby was alive up to 40* ^*th*^ *days. The problem happened to me by the doctor’s fault. She wrongly inserted her hand in to my womb.”*

### Early marriage

On average, study participants got married at the age of 14.3 years. Participants perceived that physical immaturity during marriage, pregnancy and delivery may cause of obstetric fistula. A 40-year-old woman who was married and started sexual intercourse at the age of 9 years old reported how the problem of obstetric fistula worsened from the time of marriage up to the time of delivery:

*“My urine and feaces are coming together through vagina. The problem started when I got married and started to have sex at the age of 9 years old. Initially, the problem was only passing little faeces through vagina and associated offensive smelling. When I gave the first child, the opening from anus to the vagina became wide. Now, I am suffering from the wound and offensive discharge around the genitalia.”*

### Prolonged labor

Having a prolonged and painful labor was also mentioned as a cause for obstetric fistula. For instance, a 40-year-old woman (who got married at the age of 14 years and became pregnant a year later) commented:

*“…the first pregnancy was difficult, and the labor pain stayed for five days. The problem [obstetric fistula] occurred due to long duration of labor pain. I know, I was not born with this disease. I always cry but crying cannot bring any change.” (Crying)*

### Theme 2: Health problems of obstetric fistula patients

Participants spoke about the experiences of physical health problems, social problems and psychological problems associated with obstetric fistula.

### Physical health problem of obstetric fistula

Women with obstetric fistula reported uncontrollable leakage of urine; genital sores; burning and itching of the genital area; offensive odor; leakage of faeces through the vaginal canal; wetting and soaking of their dresses, bed sheet, etc.; inability to move their body parts; and exhaustion. Several participants also described having prolonged labor pain, having still births or having lost their child shortly after delivery.

All participants suffered from uncontrollable leakage of urine and associated soaking and wetting of their clothing and bed sheets. A 35-yearold woman explained the problem as:

*“I am passing urine continuously day and night. I can’t get good sleep. My sleeping ‘****Agoza****’* (local sleeping place made of animal skin) *is always wet. Sometimes, I prefer to lie on the bare ground rather than on the ‘****Agoza****’. I would prefer to live a healthy life for five years than die having lived with this problem for 20 years.”*

Due to their obstetric fistula, women also suffered from genital sores, and burning and itching of the genital area, due to continuous dripping of urine. A 40-year-old woman with obstetric fistula described:

*“I feel burning and itching sensations around genital area. My genital area is wounded for long period of time. I take medicine for this pain frequently However, the drug reliefs the pain for short period of time.”*

Participants described feeling of exhaustion and having difficulty of moving their body parts immediately following long and painful labor.

Eight of the participants reported that their newborn died during labor and or delivery. A 35-year-old woman with obstetric fistula and history of prolonged labor explained her memory as follows:

*“I was healthy in all of my pregnancy period. But my labor pain stayed for three days. finally, I had a live birth at home, and I become unable to move my body parts at the end. The newborn died immediately though.”*

### Social health problems

Women reported experiencing social problems such as ostracization by family members, neighbors and other community members. Divorce, lack of love and support from husbands, school dropout due to discrimination by classmates, and husbands seeking second wives were other major problems reported by the participants. They also faced social disintegration and isolation, as they were not allowed to participate in social events, mourning ceremonies, weddings, ***EKUBS (****traditional social group which mainly focus on money contribution and saving and finally refunding it by lottery methods’****),*** and **MAHIBERS (***traditional social association formed for religious purpose and to help each other and poor)*.

Participants were the target of discrimination by families, neighbors, community members and others social group due to obstetric fistula and the offensive smell associated with incontinent flow of urine. A 40-year-old participant who had lived with obstetric fistula for the last 10 years explained:

*“I live with my parent following my divorces. When my mother died, my father married other women. It was difficult for me to be with my stepmother. Then, I started to live with my brother. My brother’s daughters and sons ostracize me due to an offensive smell. All family members discriminate me. If I wasn’t getting married early, I wouldn’t get this disease. Now I am living alone in a little hut.” (Crying)*

Some of the participants experienced social discrimination, which sometimes led to reciprocal hatred towards community members, resulting in disintegration. A 40-year-old woman spoke about this issue:

*“People mostly talk about me. They said that ‘this lady may not be recovered from her illnesses’. I hate to live with my neighbors. I want to live alone. But I do not know how to live and what shall I do for living?”*

Half of the participants were divorced as the consequence of obstetric fistula. Participants believed that men prefer wives who are healthy and can work and be productive. A 20-year-old woman who had been living with obstetric fistula for the last seven years stated that:

*“My husband informed that I have to sign the divorce letter. He gave some of our family property and sends me off. He also told me to have someone who could work and produce income for supporting the household. I was unable to work as he expected. Now I am working as a daily laborer. If I were healthy, he would not divorce me.” (Crying hanging down to ground)*

Participants reported that their husbands started using separate places for sleeping. Patients reported that their husbands had taken a second wife and had had children with another woman. A 35-year- old woman who presented with failure of the first surgical treatment stated:

*“My husband spends a year using a different bed at the same house. He was thinking that I would improve after a while. But he lost hopes on me and arranged a divorce letter. Nobody wants you if you become useless person like me!” (Crying)*

Another 40-year-old woman who was still living with her husband reported that her husband no longer loved her. He has a second wife and had a child with that woman:

*“Nobody knows the problem without my husband. I had never told to anybody. My husband lived with me for a long time. But he does not want to share the same bed since I am dirty to him. But we are still doing sexual intercourse. When I became ill, my husband had a second woman and they had one child. However, I should not cry today while I am in this hospital.” (Sad and Cleaning tears)*

Participants faced stigma and discrimination in school while they were attending classes. A 30-year-old woman with obstetric fistula had attended school up to grade four but was forced to drop out from school since she was unable to cope the stigma and discrimination:

*“After I developed obstetric fistula, I started education and learnt up to grade four. my classmate ostracizes me for bad smelling and flow of urine in the class. They were not sitting close to me. I used to sit far away from students. I decided to stop my education when I become unable to cope these ostracization. At the beginning of the school, I was actively learning and understand everything. Meanwhile, I started fully thinking about my problem rather than the lesson given in the class.”*

### Psychological health problems

Most of the participant perceived themselves as inferior to other healthy women. They had feelings of hopelessness with their illness or treatment and expressed feeling of fear and sadness; some had even attempted suicide. Participants felt inferior to healthy individuals since they could not control their urine and were unable to participate in socio-cultural events. Some reported that it was better to die than living with such problems. A 25-year-old woman reported that she was divorced and lives in the hermitage:

“…*I hate to live with my neighbors and my husband in the rural area. I want to leave my homeland. If I am not able to be recovered after the second surgery. I am planning to go to hermitage permanently. But I do not know how-to live-in hermitage since I am a dirty woman.”*

Most of the women with obstetric fistula reported that they were not involved in socio-cultural activities and preferred to be alone. A 25-year-old participant who had been living with obstetric fistula for the last three years shared her story:

“*As you know, it is very bad to live in holy place while I am dirty and source of bad smelling. It would be better if I die.”*

Another 40-year-old woman with obstetric fistula explained her experiences:

*“I preferred to be alone. I cannot sleep with my husband; I cannot pray in the church, I cannot sit with people in the coffee ceremony. So why do I live?”.*

Participants expressed a preference to be alone instead of being with their community members and families, fearing that they might disturb people. They reported that they became sad when they received negative responses from others. A 48-year-old woman recounted:

*“I cannot go the church to pray confidently. I cannot control my urine and flatulence. When I go to a mourning ceremony, I sit alone. I was paying to* ***senbetie Mahiber*** *(a traditional social association usually not more than 12 individuals who are preparing food and local drinks in every month by turn and celebrating together)* ***.*** *Now* ***,*** *I stopped to do so because I am afraid. I cannot feel free when people mask their mouth and nose.” (Crying)*

Most of the participants were pessimistic about their marriage and had no hope of being cured from obstetric fistula, even after second surgery. A 40-year-old woman said:

*“I am a person who is standing but not alive. If I will not be improved from this illness, I will live alone in private home. I may not able to be cured, and my problem has worsened after the operation, but others are being recovering. however, I understand one thing that darkness surrounds us despite sun keeps rising.” (crying)*

Another married participant explained her plan to divorce and living away from her residency since she is hopeless of being cured from the problem:

*“If I am not going to improve after the second surgery, I prefer to die. Otherwise I will take a small rental home at downtown and live alone and I will divorce to my husband. He is tired of caring me.” (…clearing tears from her face by towel…).*

Participants were frightened and sad as a result of peoples’ bad reaction as a result of their bad smell. A 34-year-old woman shared her experience:

*“When I approach to peoples, they cover their face and mouth by towels and hands. Then I became panic and feels shame. I felt that I shouldn’t had been born.”*

Participants had a history of suicide attempts after the onset of obstetric fistula. Two participants, 20 and 32 years old, shared their experiences:

*“I was thinking that I shouldn’t be alive as inferior of human being. I had to take some toxin drugs and die.* (Her eyes filled by tear). *After a while, I became improved to some extent; at least I could walk, eat and drink by myself following the treatment.”*

*“Even I cannot sit close to my mother and father. I tried to kill myself for several times.* (Crying…) *Once a day, my father stopped me while I tried to take rat killer (toxic substance).”*

### Theme 3: Coping mechanisms for obstetric fistula

Participants with obstetric fistula used a variety of coping mechanisms to deal with the physical health problems. These included: frequent cleaning of clothing and underwear; frequent bathing of the genital area and other body parts; use of sanitary pads; and limitation of fluid intake (to minimize flow of urine). A 40-year-ld woman shared her experiences:

*“…Previously, I was alone since I have a bad smell. Nowadays, I usually clean my body parts and washing my clothes. These practices reduce the bad smell and help me to rebuild social life.”*

Participants were concerned about the economic cost of living with obstetric fistula. A 35-year-old participant said:

*“Before attending any social event, I wash my clothes, cleaning my body. I am losing money to buy sanitary materials like soap and sanitary pads though. You know, I am not doing any business while being obstetric fistula patient.” (Crying)*

All the participants had been experiencing emotion-focused coping strategies like being alone and isolated from the community. They were afraid of leaking urine and its bad smell. A 20-year-old woman reported the condition as:

*“Other relatives and families do not ostracize me, except my husband. But I am not confident to be with them since my body smells bad.”*

## Discussion

The current study presented experiences of women living with obstetric fistula. The report contains perceived causes of obstetric fistula, and the consequences of obstetric fistula (like physical problems, social health problem and psychological difficulties) and coping mechanisms used by patients to minimize the burden of obstetric fistula.

In this study, women with obstetric fistula perceived that instrumental delivery and inappropriate care by health workers could resulted in the onset of obstetric fistula. A study conducted in Bangladesh and in Democratic Republic of Congo reported those women who delivered at health facility perceived that health care workers caused obstetric fistula by inserting sharp instruments that assist delivery [13]. Other studies on the experience of obstetric fistula survivors in Addis Ababa, Ethiopia, reported that the main causes of obstetric fistula were “being cursed”, “the will of God”, “evil spirits”, “it is because of God”, “family has done something bad to God”, “I don’t know”, and “it is my fate” as perceived by patients [14]. However, scientific finding revealed that prolonged obstructive delivery is the major cause of obstetric fistula [15].

The current finding revealed that most participants failed to notice the cause of obstetric fistula. This may cause a delay in health care seeking behaviors, thus aggravating the condition and reducing the chance of recovery. This might be due to participant’s low literacy level, and or acutely informed about obstetric fistula which might have an influence on the understanding of obstetrics fistula and its treatment options.

In the current study, some of the study participants reported that long duration of labor and early marriage were possible causes of obstetric fistula. This finding has positive implications for health care service utilization and for discouraging early marriage practices in the community.

Study participants reported that they were facing several health problems like inability to move body parts, inability to restrain urine and or faeces (resulted in bad smell), genital sores, wetting of bed sheets, and burning and itching sensations of genital area. Patients limited fluid intake to minimize these complications.

The consequences of obstetric fistula made women to feel isolated and unable to participate in socio-cultural events. They ostracized from social services, and face social disintegration, and even divorce. They also faced discrimination by family and the community members. Half of participants were abandoned by their partners. The Ethiopian Demographic and Health Survey reported that women with obstetric fistula were often socially rejected [8].

Another study on obstetric fistula patients following corrective surgery in Kenya showed that survivors were often divorced following surgical repair, even though the surgery were successful [16]. This indicates that there are maltreatment and negative attitudes in the community towards women with obstetric fistula.

Almost all participants experienced feeling of hopelessness, sadness, and inferiority associated with recurrence of surgically treated obstetric fistula. They were hopeless about their marriages and families. They were sad by their family members, relatives, classmates and community members following lack of supports. A similar study in Ethiopia reported that women with obstetric fistula had experienced anger, sadness, and shame associated with a lack of support from their husbands, family members, and community members and some of the women in that study reported suicide attempt by women before presented to the treatment center [13]. This explained that women with obstetric fistula needed special care and support from family and the community members.

In this study, participants mentioned several coping mechanisms to overcome challenges associated with obstetric fistula. They reported frequent washing and cleaning of clothes and body parts. Another study conducted in East Africa showed that all women were extremely worried about their inability to be clean and neat. They were always putting on dirty dresses, which reduced their value as women. It was not possible for them to wear new clothes, they wrapped themselves by old clothes ‘khangas’ or ‘vitenges’ (East African fabric) to prevent the continuous urine leakage [17].

Our result implies that women with obstetric fistula led difficult life. Stakeholders who are working in the area of obstetric fistula are advised to deliver awareness creation campaigns to reduce misconceptions surrounding obstetric fistula. Community mobilization programs to fight early marriage are also warranted. Focused advocacy strategies to encourage policy makers to increase the number of obstetric fistula centers in Ethiopia can also have a role in reducing the burden of obstetric fistula patients.

### Implication of the result

The current study suggested that the community has misconceptions regarding the cause of obstetric fistula. This resulted in maltreatment of community members towards obstetric fistula patients. The patients already suffering from physical and psychological health problem associated with obstetric fistula. The current situation seems victimizing the victims.

The findings of this study may also help for the health care providers who works at Hamlin Hospital to design and implement different health education and behavioral change communication strategies to increase awareness of the community about the cause obstetric fistula. Other stakeholders, which are working on the area of maternal and child health should give due emphasis to reduce the burden of obstetric fistula using multiple strategies.

Since prolonged labor mentioned as the main causes of obstetric fistula, continuous government efforts are warranted to strengthen health infrastructure near to the community to reduce the delay in reaching health facilities for delivery.

Finally, the study finding serve as baseline data for the researchers to explore perceptions and understandings of obstetric fistula from patients’ perspective. Subsequent researches which will include conducting a quantitative survey in the community and collecting information from health workers and government officials are recommended to further investigate the nature of obstetric fistula.

## Conclusion

The current study participants perceived that obstetric fistula is caused by inappropriate physical examination; inappropriate care by health workers during delivery. These misperceptions may hinder health care seeking behavior of women, especially for institutional delivery.

Obstetric fistula has several physical, psychological, and social consequences like isolated from the community members frequently divorced and lack of love and support from their partners. Consequently, they feel hopelessness and frequently attempted to suicide. Showing love and support may positively impact the quality of life of the obstetric fistula patient.

## Additional files

Additional file 1: Indepth interview Guideline (Annex 1) It is a four pages interview guide which was used for collceting data from the study particpants. It has two major sections. The first section is contain basic socio-demographic variables of the respondent. It has structured in to fifteen quastions with different levels of responses or choices. The second section contain eight major questions(with out probing questions) which assesed the lived experiences of women with obstetric fistula, in Hamlin Fistula Centre Bahir Dar, Ethiopia. (DOCX 25 kb)

Additional file 2: Themes, Catagories (Annex 2) It is a single table which shows how catagories, and themes organised during analysis. It has a title name “Showed Summary of the themes, and categories of obstetric fistula victims in Bahirdar Hamlin fistula center, Bahirdar, Amhara regional state, Ethiopia in 2016*”. (DOCX 20 kb)*

## Abbreviation

IDI: in-depth interview
