# Supplementary material for: “The sun keeps rising but darkness surrounds us”: a qualitative exploration of the lived experiences of women with obstetric fistula in Ethiopia
Source: BMC Womens Health. 2019 Feb 26;19:37. doi: 10.1186/s12905-019-0732-3 (PMC6390300; doi:10.1186/s12905-019-0732-3)
Supplement: Supplementary file 1 — Indepth interview Guideline (Annex 1) It is a four pages interview guide which was used for collceting data from the study particpants. It has two major sections. The first section is contain basic socio-demographic variables of the respondent. It has structured in to fifteen quastions with different levels of responses or choices. The second section contain eight major questions(with out probing questions) which assesed the lived experiences of women with obstetric fistula, in Hamlin Fistula Centre Bahir Dar, Ethiopia. (DOCX 25 kb) [file 12905_2019_732_MOESM1_ESM.docx]

Additional file 1

## Annex IV: Guidelines for In-Depth Interview/English Version/

1. **Socio-demographic characteristics of the respondent**

| Sr. no | Variable | Possible answers |
| --- | --- | --- |
| 1 | Participant code | ________ |
| 2 | Age (current) | ______years old |
| 3 | Age at first marriage/sexual intercourse | --------years old |
| 4 | Age at first Pregnancy/birth | --------years old |
| 5 | Circumcision | 1. Yes 2. No |
| 5 | Educational status | 1. No Education 2. Read And Wright 3. Primary Education 4. Secondary Education 5. Collage And Above |
|  | Current Marital status | - - - 1. S  1. Single 2. Married 3. Divorced 4. Widowed |
| 7 | History divorce | 1. Yes 2. No |
| 8 | Parity | __________ |
| 9 | Ethnicity | ___________ |
| 10 | Religion | 1. Orthodox   1. Muslim 2. Catholic 3. Others specify_______ |
| 11 | Employment | 1. Government 2. Private 3. Farmer 4. House wife 5. Un employed 6. Other specify__________ |
| 12 | Is the last child alive? | 1. Yes 2. No |
| 13 | Residence | 1. Rural 2. Town  - Distance in KM from this center____km |
| 14 | Delay before treatment in years | --------years |
| 15 | Number of surgeries conducted for you |  |

**II. Interview guide on lived experiences of women with obstetric fistula, Hamlin Fistula Centre Bahir Dar, Ethiopia, 2016.**

1. Could you tell me all about yourself? Probe:

- *About your current health condition?*
- *What symptoms are you feeling now?*
- *How is your condition?*
- *How do you come here for treatment?*
- *Who helped you to come here, from where you get the information about this center and their service?*
- *Can you tell me about your child hood history? Is there any association between your childhood experience and the current problem?*
- *About your marriage including the one who decided the age and the person who you married?*

1. Could you tell me about the circumstance of your pregnancy? Probes:

- *How was your history of labour and delivery when this obstetric fistula occurs?*
- *About Childbirth and what happened?*
- *Could you tell me what causes this obstetric fistula, how you become the victim of obstetrics fistula?*

1. Now let’s discus about your everyday experiences related to your health with history of obstetric fistula?Probes:

- *How do you feel about your current health situation?*
- *About physical symptoms early during theoccurrence of obstetric fistula?*
- *About physical symptoms early during the occurrence of obstetric fistula?*
- *Other related symptoms you are facing?*

1. Please tell me about your everyday experiences related to your social relation with history of obstetric fistula? Probes.

- *social relations with family and how they treat you,*
- *social relations with your husband*
- *your social network with your neighbor and relatives*
- *your social network with your social organizations (edir,ekub, others etc)*
- *Could you tell me about your finance source and how you get it?*
- *Could you tell me about problems you faced related to finance?*

1. Psychological/emotional wellbeing; could you tell me any dismay experience you faced by living with fistula? Probes:

- *Could you tell me what do you feel by living with obstetric fistula?*
- *Could you tell my any depression associated with fistula?*
- *Could you tell me in detail your experience related to loneliness, depression,etc?*

1. How have you been able to cope with the condition? Probes:

- *Could you tell me your experience of coping with these all problems starting from the occurrence of obstetric fistula up to now?*
- *What and How do you do to cope with your physical health problems?*
- *What and How do you do to cope with social problems attributed from the problem?*
- *What other coping mechanism do you have?*
- *How much these copying mechanism helps you?*

1. Is there anything that I shouldn’t ask you but important for this research?
2. Do you have anything to Add?

Thank you

Date ________________________signature _______________________________
