# Supplementary material for: “The sun keeps rising but darkness surrounds us”: a qualitative exploration of the lived experiences of women with obstetric fistula in Ethiopia
Source: BMC Womens Health. 2019 Feb 26;19:37. doi: 10.1186/s12905-019-0732-3 (PMC6390300; doi:10.1186/s12905-019-0732-3)
Supplement: Supplementary file 2 — Themes, Catagories (Annex 2) It is a single table which shows how catagories, and themes organised during analysis. It has a title name “Showed Summary of the themes, and categories of obstetric fistula victims in Bahirdar Hamlin fistula center, Bahirdar, Amhara regional state, Ethiopia in 2016”. (DOCX 20 kb) [file 12905_2019_732_MOESM2_ESM.docx]

**Additional file 2**

**Annex 2**

Showed Summary of the themes, and categories of obstetric fistula victims in Bahirdar Hamlin fistula center, Bahirdar, Amhara regional state, Ethiopia in 2016

| **Themes** | **Categories** |
| --- | --- |
| **Perceived cause of Obstetrics Fistula** | *Instrument assisted delivery, inappropriate physical examination and care, early marriage, Prolonged labor* |
| **Health Problem of Obstetrics Fistula patients** |  |
| **Physical health problem of OF**    **Social Health problems OF**  **Psychological Health Problems OF** | *Urine incontinence*, *Genital sore and burning pain,* being exhausted following labor pain, |
|  | *Ostracization/Discrimination,* *Divorce,* *Lack of love and support by husbands,* *School dropout* |
|  | *Feeling of inferiority,* *Social Isolation,* *Feeling of Hopelessness,* *Fear of people’s response,* *Suicide Attempt* |
| **Coping Mechanisms of Obstetrics Fistula** |  |
|  |  |
|  | *frequent cleaning of clothing, frequent personal bathing, limitation of fluid intake, Isolation* |

^OF= obstetrics fistula^
